# A new approach for elimination of gastric cancer deaths in Japan

**DOI:** 10.1002/ijc.27965

**Published:** 2013-03-15

**Authors:** Masahiro Asaka

**Affiliations:** 1 Department of Cancer Preventive Medicine, Hokkaido University Graduate School of MedicineKita-Ku, Sapporo, Japan

**Keywords:** gastric cancer prevention, *H. pylori* eradication, endoscopic surgery, early gastric cancer

## Abstract

We explore a strategy for the elimination of gastric cancer deaths in Japan. Most gastric cancer is due to *H. pylori* infection in Japan. The effect of *H. pylori* eradication therapy on gastric cancer prevention in younger people is excellent, but it declines along with advancing age. Therefore, a test-and-treat approach to *H. pylori* infection is recommended in younger people, while for people aged 50 years or older a combination of countermeasures for *H. pylori* eradication that includes primary prevention and secondary prevention by endoscopic examination will reduce gastric cancer deaths, since this method will increase early detection if the disease occurs. In this paper, I described a new strategy of elimination of gastric cancer deaths in Japan due to such a high quality of diagnosis and treatment for gastric cancer. If this strategy succeeds, the incidence of gastric cancer in Japan may decrease much longer than 10 years.

Gastric cancer is the second most common cause of cancer deaths worldwide.^1^ Until the early 20th century, Europe and the United States suffered a high incidence of gastric cancer. The 20th century also witnessed that the incidence of gastric cancer rapidly decreased coincidently with the changes in life style, sanitation and the widespread adoption of refrigeration for food preservation. Currently, three East Asian countries, Japan, China and Korea, account for about 60% of new gastric cancers. Early studies of the possible cause of gastric cancer emphasized dietary factors such as excessive intake of salt or nitrates and hereditary factors. The culture of *Helicobacter pylori* (*H. pylori*) in 1983^2^ resulted in research focused on proving the causal relationship between *H. pylori* infection and gastritis and gastric cancer. As a result, in 1994, *H. pylori* was classified as a definite carcinogen by the International Agency for Research on Cancer (IARC) of the World Health Organization.^3^ Since that time, many clinical studies have been conducted to examine how eradication of *H. pylori* might contribute to the prevention of gastric cancer. Because of a low incidence of gastric cancer, the relatively short duration of available studies and the general lack of risk stratification, statistically significant decreases were infrequently reported. In 2008, a multicenter clinical study was conducted in Japan to examine the incidence of new gastric cancer after endoscopic mucosal resection in high-risk patients with early gastric cancer who were randomly allocated to eradication of *H. pylori* group.^4^ The study showed that *H. pylori* eradication resulted in a reduction in the incidence of new gastric cancers by approximately one-third, thus demonstrating the efficacy of *H. pylori* eradication in reducing the incidence of gastric cancer. The study also confirmed that eradication could not completely prevent metachronous gastric cancers and that periodic follow-up for gastric cancer would be required even after eradicating *H. pylori* in high-risk patients.

In Japan, gastric cancer screening has long been done by using barium contrast images.^5^ However, evidence that *H. pylori* infection played an important role in the development of gastric cancer and that *H. pylori* eradication could prevent or reduce the incidence of gastric cancer suggested the need for a new strategy to eliminate gastric cancer in Japan. The new strategy could be one that combined primary prevention by *H. pylori* eradication with secondary prevention using surveillance of high-risk patients. Japan is currently at the forefront of devising methods and procedures for the elimination of gastric cancer based on the current high level of knowledge and technology and experience in gastric cancer screening. Here, we briefly describe the history of gastric cancer prevention in Japan and introduce the changes in *H. pylori* therapy and a new program that combines primary prevention and secondary prevention that will be soon introduced in Japan.

## Previous Preventative Measures for Gastric Cancer in Japan

The prevention of cancer, including gastric cancer, has primarily focused on secondary measures for early detection of cancer, rather than on primary prevention aimed at elimination of the causes in Japan. Indirect barium contrast imaging has been used as the screening method for gastric cancer; however, despite the long interest and emphasis, the screening rate was only 9.6% in 2010.^6^ Screening for gastric cancer based on barium contrast imaging also does not have a high sensitivity for detecting early cancer^7^ and is associated with considerable exposure to radiation. Furthermore, targeting all people aged 40 years or older for screening is a major problem as people aged below 50 years account for only about 3% of all patients with gastric cancer in Japan.^6^, ^8^ Moreover, *H. pylori*-negative patients with minimal or no atrophy of the gastric mucosa are very unlikely to develop gastric cancer,^9–11^ and thus, these patients are unlikely to benefit from annual barium contrast screening and are still exposed to the adverse effects of radiation.

The most serious disadvantage with Japan's attempts to prevent gastric cancer was the inability to implement primary prevention, which is understandable as the cause of gastric cancer had not been identified in the 1970s when programs of screening for this cancer were begun. However, we now know that more than 95% of gastric cancers are due to *H. pylori* infection in Japan and Korea.^10^, ^11^ As a general rule for cancers caused by infections, such as liver cell cancer and cervical carcinoma, primary prevention based on preventing the infection or early eradication before significant damage is done is preferred over screening (*i.e.*, primary prevention is superior to secondary prevention). Primary preventative measures for gastric cancer have yet to be started in Japan, and Japan has relied on barium contrast screening for 30 years. A decrease in the age-specific mortality rate of gastric cancer has been experienced from 1970 to 2010 in both sexes in Japan. However, this seems most likely to reflect the decrease of incidence rate of gastric cancer known to have occurred in both sexes in Japan during the same period.^8^ The aging of the population has increased the population at risk, and thus the number of patients dying from gastric cancer has remained unchanged at around 50,000 per year.^12^ The lack of a reduction in the total number of deaths despite the decline in age-standardized mortality rates provided important evidence to the Japanese Government that current programs were not effective in the prevention of gastric cancer deaths.

## Effect of *H. pylori* Eradication on Gastric Cancer Incidence

Intervention studies that assessed the preventative effect of *H. pylori* eradication on gastric cancer have been conducted in healthy individuals worldwide.^13–17^ In the United States and Europe, however, most studies were terminated before enrolling enough subjects for significant analysis because the incidence of gastric cancer is extremely low in these countries.^18^, ^19^ Overall, the annual incidence of gastric cancer has been reported to be only 0.1–0.3%^20^, ^21^ in persons infected with *H. pylori.* In contrast, the annual incidence of metachronous recurrence is reported to be in the range of 3–5% of patients who have undergone endoscopic surgery to remove early gastric cancer.^22^, ^23^ Our study evaluated recurrence of metachronous gastric cancer in 544 patients who had received endoscopic treatment. They were randomly allocated to *H. pylori* eradication or noneradication groups and were followed up with annual endoscopic examinations for 3 years. As a result, metachronous recurrence was found in nine and 24 subjects from the eradication and noneradication groups, respectively. The eradication group had a significantly lower incidence of metachronous gastric cancer with a hazard ratio of 0.339 according to intention-to-treat analysis.^4^

A large-scale cohort study was reported from Taiwan, in which about 80,000 patients with peptic ulcer were followed up for 10 years after *H. pylori* eradication therapy.^24^ The patients were assigned to an early eradication group (patients underwent *H. pylori* eradication therapy at the time of diagnosis) or a late eradication group (patients underwent *H. pylori* eradication therapy at 1 year after diagnosis). As a result, the incidence of gastric cancer was markedly lower in the early eradication group than in the late eradication group (*p* < 0.02). This study is important in showing that while the effect of *H. pylori* eradication therapy in reducing the incidence of gastric cancer is obvious, earlier eradication can be more effective. These studies also suggest that *H. pylori* has a cancer promotion effect over and above its ability to cause atrophic gastritis. In 2011, a possible mechanism was described due to incomplete repair of genes damaged by *H. pylori* infection, which cleaves double-stranded DNA in the nuclei of gastric epithelial cells.^25^ In addition, they demonstrated that the genetic defect remained as long as *H. pylori* infection persisted, further supporting the importance of *H. pylori* eradication in high-risk patients.

## Gastric Cancer Elimination Project

It has been demonstrated that most gastric cancer is due to *H. pylori* infection (*i.e.*, a necessary but not sufficient cause), and we believe it is time for a major strategic shift in the preventative measures for gastric cancer. Preventative measures for liver cancer have been conducted with the focus on hepatitis viruses since 2002 in Japan, and this has succeeded in decreasing the mortality.^26^, ^27^ In marked contrast, annual deaths from gastric cancer have remained at around 50,000 for the last few decades, suggesting that the current preventative measures have been less than satisfactory.^8^ Even though viruses and bacteria are not the same, completely different preventive measures should not be taken for liver cancer and gastric cancer when both are caused by infection. In 2012, the section on Current Status of the Basic Plan to Promote Cancer Control Programs of the Japanese Government issued a new plan to determine Cancer Control Programs for next 5 years in Japan, including those caused by microorganisms such as human papillomavirus associated with the development of cervical carcinoma, hepatitis viruses associated with liver cancer, human T-cell leukemia virus Type I associated with adult T cell leukemia and *H. pylori* associated with gastric cancer. For *H. pylori*, the benefits of bacterial eradication should be examined based on findings from Japan and abroad.^28^

Meanwhile, the Japanese Society for Helicobacter Research published a guideline recommending that all *H. pylori*-infected people receive bacterial eradication therapy.^29^ In response to this, the Japanese government has expanded coverage by the national health insurance scheme. In addition to gastroduodenal ulcer, three other indications for *H. pylori* treatment including mucosa-associated lymphoid tissue (MALT) lymphoma, postendoscopic surgery for early gastric cancer and idiopathic thrombocytopenic purpura (ITP) have been newly designated. Japanese insurance coverage for *H. pylori* eradication therapy for an indication other than gastroduodenal ulcer is the first in the world.

Currently, the Japanese Minister of Health, Labour and Welfare has been asked to extend insurance coverage to chronic gastritis by the presidents of the Japanese Society of Gastroenterology, the Japan Gastroenterological Endoscopy Society and the Japanese Society for Helicobacter Research, raising expectations that approval of the request will be granted, as the final target to eradicate gastric cancer is to eliminate chronic gastritis due to *H. pylori* infection. The Japanese medical insurance is a universal health insurance system covering all citizens with freedom of choice of medical institution and high-quality services with low costs.^30^ This insurance covers 90% of payment of medical expenses in persons aged above 75 years, 80% of payment at 70 to 74 years and 70% of payment less than 69 years. We are currently negotiating with the Japanese Government to expand the application of medical insurance to chronic gastritis. Hopefully, this will be approved in 2013 for patients with *H. pylori*-related chronic gastritis with endoscopy used to confirm the diagnosis of chronic gastritis.

**Figure 1:**
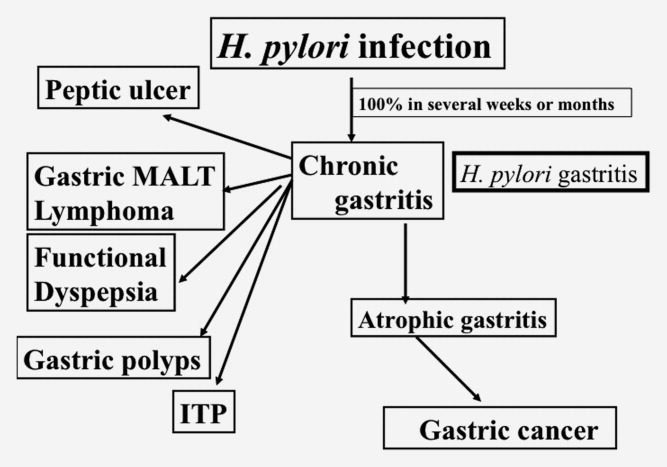
Progress of *H. pylori* infection. *H. pylori*-related chronic gastritis is leading to peptic ulcer, gastric MALT lymphoma, functional dyspepsia, gastric polyps, ITP, atrophic gastritis and gastric cancer.

**Figure 2:**
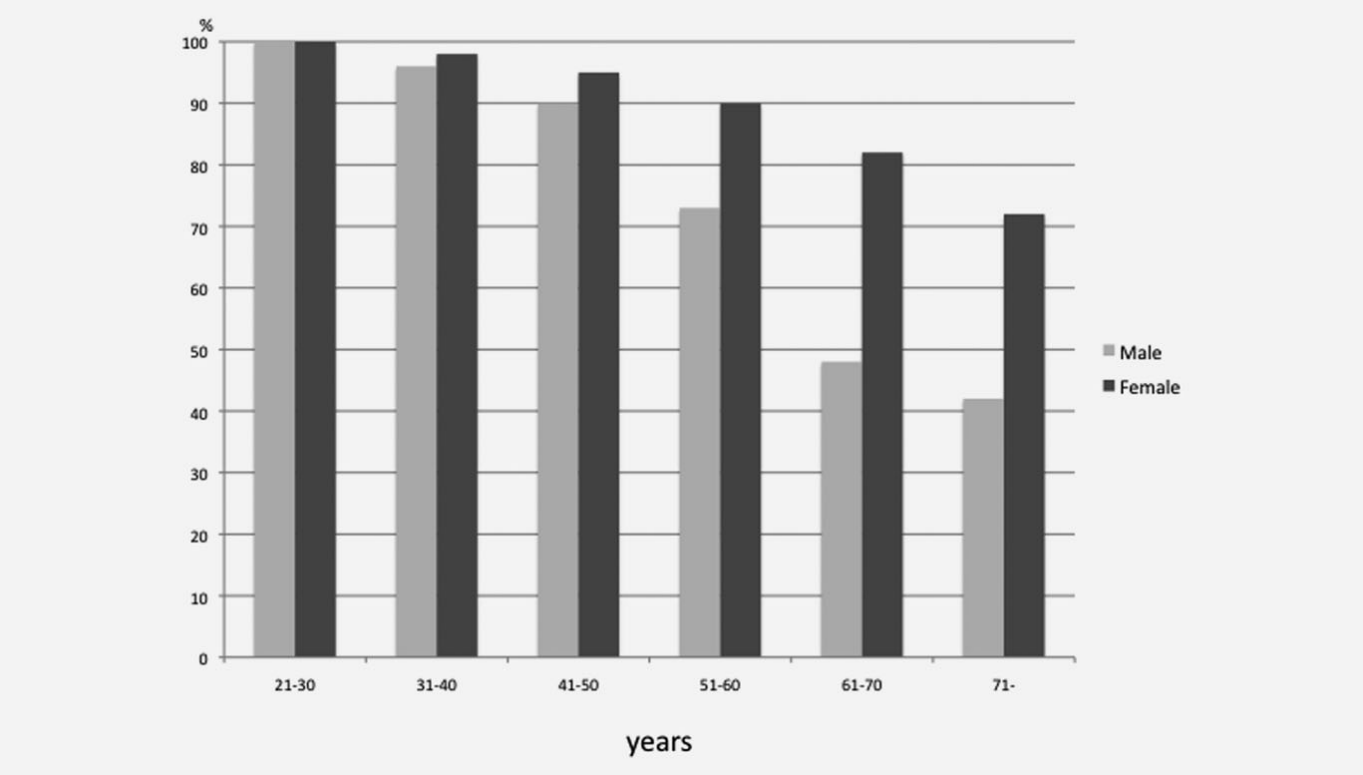
Possible rate of gastric cancer prevention by eradication of *Helicobacter pylori*.^31^

**Figure 3:**
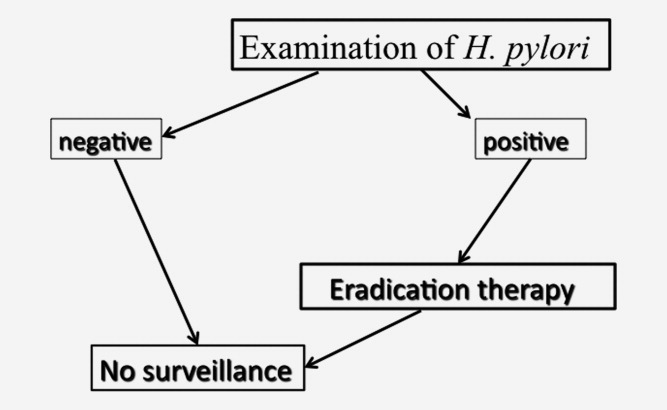
An approach for elimination of gastric cancer deaths in younger generation (before 20 years) in Japan.

When *H. pylori* eradication therapy for chronic gastritis is covered by national health insurance, different measures should be taken for people aged below 20 years and people aged 50 years or older. Bacterial eradication in persons aged below 20 years may achieve prevention of diseases such as peptic ulcer, gastric MALT lymphoma, functional dyspepsia, gastric polyps, ITP, atrophic gastritis and gastric cancer associated with *H. pylori*-related chronic gastritis (Fig. 1). We reported that incidence of gastric cancer after eradication of *H. pylori* increases along with advancing age (Fig..2).^31^ Thus, a test-and-treat approach is recommended for younger people that includes universal *H. pylori* testing and immediate bacterial eradication in those with a positive result (Fig..3).

Because people aged 50 years or older frequently have atrophic gastritis and are likely to be at risk for cancer despite *H. pylori* eradication, we recommend that they will be referred for evaluation of the presence and severity of their *H. pylori*-related gastritis. Those with *H. pylori* infection should receive endoscopic examination (which will be covered by Japanese medical insurance) to evaluate for the presence and severity of atrophic gastritis. If people have a family history of gastric cancer and/or have been diagnosed as having atrophic gastritis by previous endoscopic examination, additional endoscopic examinations will also be offered in cases without *H. pylori* infection.

**Figure 4:**
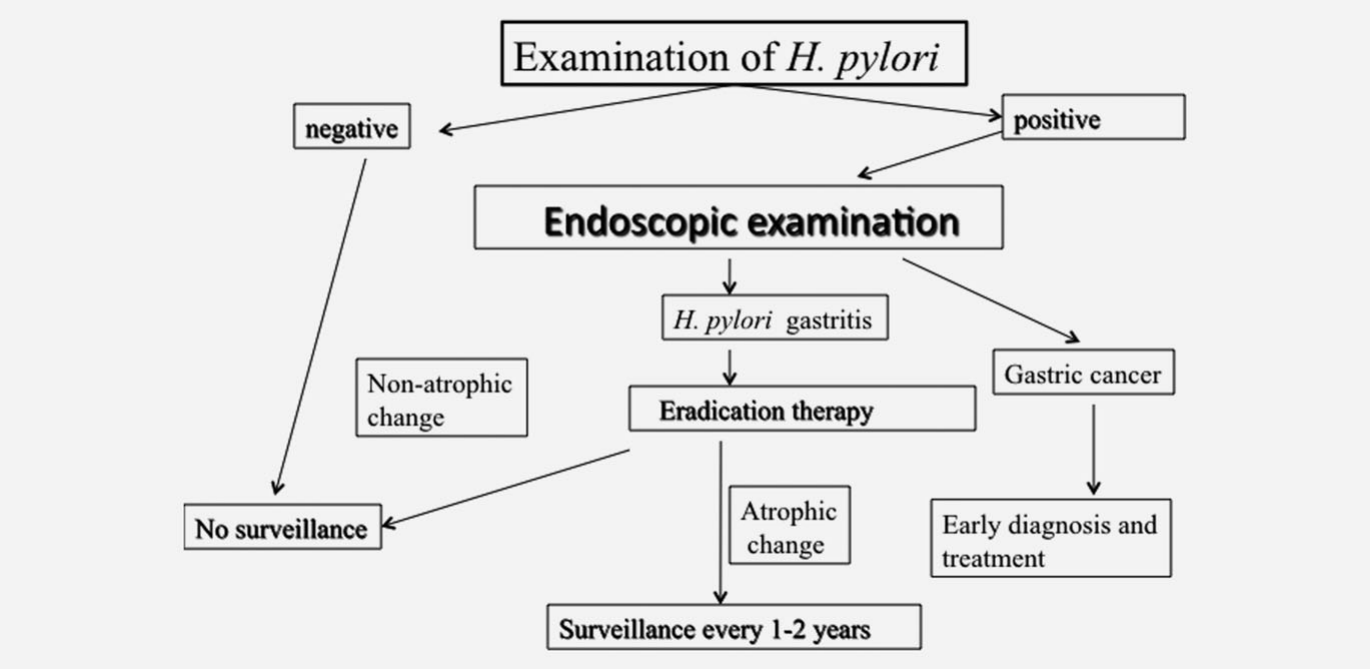
An approach for elimination of gastric cancer deaths after 50 years in Japan.

We expect that many patients with gastric cancer will be discovered during this endoscopic examination. This project thus includes a form of endoscopic screening supported by medical insurance. Those without gastric cancer should receive bacterial eradication therapy. Persons whose endoscopic examination shows findings close to normal can be transferred to a no-surveillance group. If atrophic gastritis is found in people, a repeat endoscopic examination should be performed 1 to 2 years later and they should be considered for a surveillance program; the frequency and nature of which will depend on the results of ongoing and subsequent research on surveillance based on risk stratification (Fig. 4). As described above, the program combines primary prevention (*H. pylori* eradication) and surveillance with early cancer detection for those remaining at risk for development of gastric cancer despite *H. pylori* eradication.

## Financial and Social Effects of Gastric Cancer Elimination

The cost of gastric cancer treatment in Japan is currently around 300 billion yen per year and will exceed 500 billion yen annually if measures are not taken for a decade or so. However, if the incidence of gastric cancer is reduced by *H. pylori* eradication, medical costs should be lowered substantially.^31^

Another important issue, in addition to cost, is the effect on society. By periodical follow-up endoscopy after *H. pylori* eradication therapy, most gastric cancer can be detected at an early stage, resulting in a quite favorable prognosis and a sharp decrease of gastric cancer-related deaths. Potentially, it might be possible to eliminate gastric cancer-related deaths from Japan around the middle of this century.

The principle of “innocent until proven guilty” is accepted in the legal field. Conversely in the field of infectious diseases, the principle of “guilty until proven innocent” applies. Therefore, proactive preventive measures are used for cancers that are suspected to be caused by infection so that the incidence of the target infection is dramatically decreased, thereby resulting in a reduction of cancer-related deaths. The effect of primary prevention based on the causes of cancer is more reliable and durable than secondary measures including screenings, and it also helps to reduce medical costs.

The possible success with elimination of gastric cancer in Japan should lead other countries with a high incidence of gastric cancer such as East Asia and Latin America to consider using a similar strategy, which might then lead to extermination of gastric cancer worldwide.

## Conclusion

A gastric cancer elimination project that combines *H. pylori* eradication therapy and surveillance of high-risk patients is both appropriate and feasible for Japan, where excellent methods of diagnosis and endoscopic treatment for early gastric cancer are already available. In this country, the baby-boom generation is now passing 60 years and reaching the cancer-prone age, and therefore, an increase of medical costs related to gastric cancer is impending. Application for medical insurance in patients with *H. pylori*-related chronic gastritis due to the Basic Plan to Promote Cancer Control Programs might be a first step to eliminate gastric cancer deaths in Japan.
